# Supplementary material for: Epidemiological clinical profile and closure of chronic plantar ulcers in patients with leprosy sequelae undergoing orthopedic surgery in a municipality in western Amazon
Source: PLoS One. 2023 Jul 28;18(7):e0284706. doi: 10.1371/journal.pone.0284706 (PMC10381085; doi:10.1371/journal.pone.0284706)
Supplement: Attachment — Submitted filename: Response to Reviewers Plos One.docx [file pone.0284706.s002.docx]

**RESPONSE TO REVIEWERS**

PONE-D-22-04569

EPIDEMIOLOGICAL CLINICAL PROFILE AND CLOSURE OF CHRONIC PLANTAR ULCERS IN PATIENTS WITH LEPROSY SEQUELAE UNDERGOING ORTHOPEDIC SURGERY IN A CITY IN WESTERN AMAZONIA

**Journal Requirements:**

When submitting your revision, we need you to address these additional requirements.

1. Please ensure that your manuscript meets PLOS ONE's style requirements, including those for file naming. The PLOS ONE style templates can be found at

https://journals.plos.org/plosone/s/file?id=wjVg/PLOSOne_formatting_sample_main_body.pdf and

https://journals.plos.org/plosone/s/file?id=ba62/PLOSOne_formatting_sample_title_authors_affiliations.pdf

***Response: We fully reviewed the manuscript and followed the instructions according to Guidelines.***

2. Please provide additional details regarding participant consent. In the ethics statement in the Methods and online submission information, please ensure that you have specified (1) whether consent was informed and (2) what type you obtained (for instance, written or verbal, and if verbal, how it was documented and witnessed). If your study included minors, state whether you obtained consent from parents or guardians. If the need for consent was waived by the ethics committee, please include this information.

If you are reporting a retrospective study of medical records or archived samples, please ensure that you have discussed whether all data were fully anonymized before you accessed them and/or whether the IRB or ethics committee waived the requirement for informed consent. If patients provided informed written consent to have data from their medical records used in research, please include this information.

***Response: We emphasized in the Materials and Methods section that all participants signed an Informed Consent Form (page 6, lines 133 and 134).***

At this time, please address the following queries:

a)	Please clarify the sources of funding (financial or material support) for your study. List the grants or organizations that supported your study, including funding received from your institution.

b)	State what role the funders took in the study. If the funders had no role in your study, please state: “The funders had no role in study design, data collection and analysis, decision to publish, or preparation of the manuscript.”

c)	If any authors received a salary from any of your funders, please state which authors and which funders.

d)	If you did not receive any funding for this study, please state: “The authors received no specific funding for this work.

***Response: The author(s) received no specific funding for this work.***

Additional Editor Comments (if provided):

The methods part shall be more detailing to express the how you complete the experiment.

***Response: The Materials and Methods section was fully updated in order to improve reading and chronologically organize the steps followed, including the recruitment criteria (pages 5-8, lines 117 to 144 and 153 to 169).***

**Reviewer #1:** The authors sought to describe the clinical, epidemiological profile and the results of orthopedic surgical treatment of chronic and relapsing foot ulcers in leprosy patients, the purposes of this study seem to be clear, however, several serious issues must be addressed, and it is suggested that the authors should consider all these comments before submitting it for peer review.

1. For the abstract session, the authors should briefly explain the significance of this study before giving the purposes; more details about the methods and main findings of this study should be presented here.

***Response: The abstract was rewritten in order to attend all suggestion given by the three reviewers (page 2 – lines 24 to 43).***

2. For the introduction session, I could not see the gap that the authors were going to bridge, nor can I see the novelty of this study. Did the authors aim to verify the effects of the surgical orthopedic treatment proposed by Hidalgo and Shaw in the last century (1986)?

Numerous studies have investigated the novel surgical treatments for plantar ulcers in leprosy, thus what are the main novelty and significance of this study? It is suggested that the authors must consider making a throughout review of previous studies and consider rewriting this part to further highlight the novelty and significance;

***Response:*** ***Regarding the description of the technique used, there was a mistake: in fact, the technique used was that of Srinivasan and not of Hidalgo and Shaw, as previously described.***

***Regarding the literature review, the following text was added (included in the Introduction section on pages 4- 5, lines 87 to 110):***

***“We found in the medical literature several alternatives for the management of chronic plantar ulcer from conservative to surgical treatment. Conservative treatment ranges from the local use of a variety of antibiotics, antiseptic agents, chemical, biological and vegetal extracts; use of vasodilators, autolytic and biochemical debridements, growth factors, total contact plaster and customized therapeutic insoles and footwear among other dressing forms and products [16-23].***

***However, studies by Noordeen and Srinivasan demonstrated that chronic plantar ulcers with bony prominences associated with an inflammatory process between the friable tissue and the bone and which were treated conservatively by dressings, rest or total contact plaster, recur around one month after healing [24].***

***Due to the high rate of recurrence of these lesions by conservative methods, several surgeons around the world have reported their techniques with their advances and limitations. Methods such as: a) surgical debridements [22, 25, 26]; b) use of skin grafts from the dorsum of the foot [27]; c) posterior tibial nerve neurolysis [28, 29]; d) use of local bipedicled flaps [30]; e) plantar flaps with blood and neurological plexus supply [25, 30, 31].***

***It is important to emphasize that the several surgical techniques to close plantar ulcers do not contemplate the correction of plantar bone fragments that are predisposing factors for the appearance of plantar ulcers as well as their recurrence [7, 11, 30, 31].***

***We found that these techniques consist primarily in the debridement of infected tissues and the closure of the ulcer pure and simple. The bipedicle flap technique proposed by Srinivasan mobilizes the flap around the ulcer and was the technique used by the author differently from the techniques presented, the author associates the technique with plantar bone resection in cases where there is presence of these bone lesions (identified in the foot radiographs in the anteroposterior and profile views).”***

2.2) the authors should consider further strengthening the logical structure of this part, making clear explanations of the definition, symptoms, treatments of foot ulcers in leprosy patients;

***Response: This review was done in detail and are described in the restructured paragraphs of the introduction (page 4, lines 80 to 86):***

*“****Price in 1964 introduced the term "perforating ulcer" as a chronic ulceration of an anesthetic region of the foot related to areas of bony prominences, resistant to local or systemic treatment and characterized by a high tendency to recur [11].***

***Srinivasan defines chronic ulcer as the ulcer that does not heal easily due to two main causes: a) presence of deep and persistent infection or b) the presence of plantar bony prominences due to disintegration of bony framework caused by neurological injury. Areas of ischemia caused by bony prominences are factors of non-healing [10,15].”***

3) update the statistical data and their corresponding refs in this part.

***Response: The epidemiological profile was updated with data from 2021 and 2022. These informations were added to the manuscript on page 3, lines 57 to 68.***

***“The World Health Organization (WHO) has defined leprosy as a public health problem, especially in countries whose prevalence rate exceeds one case per 10,000 inhabitants. In the year 2020, about 127,396 new cases of the disease were reported in the world, of these, 19,195 (15.1%) occurred in the Americas region, with Brazil with 17,979 cases corresponding to 93.6% of the number of new cases in the Americas [3,5].***

***The neurological lesion determines sensory and motor alterations that lead to the installation of varying degrees of physical disabilities (WHO-DG), among them are the plantar ulcers that due to the difficulty in healing these lesions constitute a major challenge among health professionals who deal with this issue [6-8].***

***Plantar ulcers are the most common severe disabilities due to leprosy and occur in approximately 10 to 20% of all patients affected by the disease, despite recent advances in multidrug therapy [9-11].”***

3. for the method section, the author must explain the method in detail. For example,

3.1) no details about the subject recruitment criteria;

***Response: The Materials and Methods section was fully updated in order to improve reading and chronologically organize the steps followed, including the recruitment criteria (pages 5-8, lines 117 to 144 and 153 to 169).***

3.2) no details about the clinical profile used for evaluation in this study;

***Response: Clinical profile for evaluation of recruitment were included.***

***Pages 5-6, lines 123 to 137***

***“Patient population and study area***

***Patients with chronic plantar ulcer with presence of bony prominences in the radiographic study of the foot of leprosy origin seen at the Policlínica Antônio Aleixo and submitted to orthopedic surgical treatment at Adriano Jorge Foundation Hospital.***

***Patient recruitment and selection***

***Polyclinic Colônia Antônio Aleixo***

***Patients over 18 years of age, of both genders, who presented chronic plantar ulcers unresponsive to conventional dressings, with presence of bony prominences in the bed of the lesion identified by X-ray in the anteroposterior (AP) and profile (P) incidence were eligible for inclusion in this work.***

***These patients were invited to participate by filling out the Informed Consent Form (ICF) and at that moment the following preoperative exams were requested: blood count, coagulogram, urea, creatinine, sodium, potassium, glycemia, electrocardiogram, and AP/P chest X-ray. These exams are taken to the cardiologist in the same unit for the preoperative risk.”***

3.3) it is hard to understand what the authors tried to explain in the two paragraphs below Figure 1. These contents were also certainly not details about the patient recruitment and selection;

***Response: Below Figure 1, we included the specifications of the local health services where the patients were transferred after the surgical procedure (page 7, lines 153 to 163).***

3.4) it is suggested that the authors should further explain the statistical methods applied for this study.

***Response: Statistical analysis was revised and rewritten. The correction is found on page 8, lines 170 to 181).***

4. For the results session,

4.1) it is suggested that the authors should include some sub-sessions here for better description;

***Response: We organized chronologically the results obtained as well as kept only one table with all the variables and the p-values (pages 8-10, lines 188 to 222).***

4.2) the results are duplicates in the results session, on the tables, and in the discussion session. I recommend the authors keep just the table on the results table and make the discussion stronger. The text on the results session with the description of the table is not helping the reader.

***Response: We excluded Tables 1 and 2, that indeed were duplicates and kept all the relevant informations in Table 1 (page 10).***

5. For the discussion session, few deep comparisons were made, and no practical information was presented based on the findings of this study. It is suggested that the authors should make more comparisons with previous related studies and explain the limitations of this study.

***Response: In order to readjust and provide new references for the Discussion section, we made a major revision on this section, as shown on pages 11-14, lines 224 to 323.***

6. For the conclusion session, it is suggested that this part should be further strengthened based on the main findings of this study.

***Response: The Conclusion was rewritten as follows:***

***“In the present study, it was observed that patients with chronic plantar leprosy ulcers are mostly male, with a mean age of 69.5 years.***

***The population has low education levels and limited family and social ties, reinforcing the stigma and isolation of these individuals. The period of permanence of the opening of the chronic plantar ulcer was mostly above 10 years, ranging from 0 to 50 years. It was also noted that most of the patients evaluated did not make regular use of insoles and adapted footwear.***

***Finally, it was observed that surgical treatment using the local bipedicle skin flap technique associated with resection of bony prominences and corrective osteotomies is an effective therapeutic method for the closure of chronic plantar ulcers. It is a reproducible method with excellent results.”***

**Reviewer #2:** Review comment

This manuscript entitled “Epidemiological Clinical Profile and Closure of Chronic Plantar Ulcers in Patients with Leprosy Sequelae Undergoing Orthopedic Surgery in a City in Western Amazonia” primarily aimed to investigate the epidemiological profile of patients with chronic plantar ulcers with difficulty in healing, secondary to leprosy. The results of this study provide guidance for public health and clinical medicine. While it is a very interesting topic. But I think this manuscript has some flaws to fill in before it can be published in a journal. There are several questions should be addressed, which list below. I give a minor revision for this manuscript.

Specific comments

1. In the Abstract part, in the opinion of reviewer, the author provided too much descriptions of background, which may be too long-winded. I suggest that the authors provide more descriptions of conclusion of this study in the abstract part.

***Response: The Abstract section has been rewritten to address this request of improvement. It is shown on page 2, lines 24 to 46.***

2. “A total of 234 patients (295 feet) underwent surgical treatment of ulcers: 129 (55.1%) were male; mean age was 69.5 years.” Please provide more anthropometric information about these patients, such as Height, weight, length of illness.

***Response* *Although we find it relevant and important to include the anthropometric measurements so pertinently suggested, it was not the scope of our study. We includeded this as one of the limitations of our work (page 14-15, lines 325 to 329).***

3. In the introduction part.“It may present slow and progressive evolution, leading to deformities and physical disabilities, often irreversible.” Please add some references to support this sentence.

***Response: Addressing this suggestion, we included the following references (page 3, lines 54 to 56)***

1. ***Souza CDF, Fernandes TRMO, Matos TS, Ribeiro-Filho JM, Almeida GKA, Lima JCB et al. Degree of physical disability in the elderly population affected by leprosy in the state of Bahia, Brazil. Acta Fisiatr. 2017; 24(1): 27-32.***
2. ***Barreto JG, Bisanzio D, Frade MAC, Moraes TMP, Gobbo AR, Guimarães LS et al. Spatial epidemiology and serologic cohorts increase the early detection of leprosy. BMC Inf Dis. 2015; 15(1): 1-9.***

4. “Nerve damage caused by Mycobacterium leprae induces sensory and motor alterations that lead to the installation of varying degrees of a physical disability that can interfere with patients’ social and economic life, resulting in stigma and discrimination.” This sentence is too general and unclear. Can you be more specific about what you mean for nerve damage.

***Response: Page 3, lines 62 to 65.***

***“The neurological lesion determines sensory and motor alterations that lead to the installation of varying degrees of physical disabilities (WHO-DG), among them are the plantar ulcers that due to the difficulty in healing these lesions constitute a major challenge among health professionals who deal with this issue [6-8].”***

5. “This change in the foot bone arrangement then causes a maladjusted gait in the patient, leading to new pressure points in non-appropriate parts of the foot.” Which parts of the foot.

***Response: We included this specific information on page 3, line 66 to 68. The parts of foot are as follows: forefoot, midfoot, hindfoot***

6. “These lesions must be addressed and adequately prevented, as they constitute entry points for infections that can progress to severe complications, such as osteomyelitis, plantar ulcers, and even limb amputations.” Please use more than one references to prove this statement.

***Response: The following references were added to support the information:***

1. ***Batista KT, Monteiro GB, Y-Schwartzman UP, Aureliano A, Rosa AG, Correia CZ, et al. Treatment of plantar ulcer due to leprosy. Rev. Bras. Cir. Plast. 2019; 34: 497-503.***
2. ***Tashiro S, Gotou N, Oku Y, Sugano T, Nakamura T, Suzuki H, et al. Relationship between plantar pressure and sensory disturbance in patients with Hansen's disease-Preliminary research and review of the literature. Sensors (Basel). 2020; 20(23): 6976.***

7. What is the author's research hypothesis, which I suggest to be added to the last paragraph of the introduction?

***Response: Orthopedic surgical treatment of trophic plantar ulcers with resection of bony prominences and corrective osteotomies, associated with a skin flap, is effective for the complete closure of these lesions.***

8. In the Methodology part.” After healing, new radiographs were taken to control and request the manufacture of customized insoles and footwear, aiming at accommodating and protecting the feet.” What is the basis for the customization of insoles and footwear?

***Response: Page 8, lines 168.***

***Customization is based on complete closure of the plantar ulcer and the patient is advised by the physician to visit the orthopedic workshop for analysis of pressure points through the podoscope to determine the customization of the insole and specific footwear for each patient.***

9. In the Results part. In the opinion of the reviewer, the pixel in Figure 1 is too blurred, please replace a clearer figure.

***Response:*** *The pictures of Figure 1 were replaced (page 7).*

10. In the Discussion part. What are the limitations of this study? Please provide relevant description.

***Response: Study Limitations section is shown on page 14, lines 325 to 330.***

***“We emphasize that this study has limitations where there is a need for a second evaluation in the search for long-term results obtained from the surgical intervention performed and its relationship with the indices of quality and lifestyle of patients, personal satisfaction as well as the recurrence rate of lesions. For this, more anthropometric information about these patients, such as height, weight, length of illness.”***

11. In the Conclusion part. In the opinion of the reviewer, the description in the conclusion part was too verbose, and the reviewer suggests that the authors should abbreviate the section and focus on the main findings of this study.

***Response: Conclusion section was rewritten and is shown on page 15, lines 333 to 343.***

***“In the present study, it was observed that patients with chronic plantar leprosy ulcers are mostly male, with a mean age of 69.5 years.***

***The population has low education levels and limited family and social ties, reinforcing the stigma and isolation of these individuals. The period of permanence of the opening of the chronic plantar ulcer was mostly above 10 years, ranging from 0 to 50 years. It was also noted that most of the patients evaluated did not make regular use of insoles and adapted footwear.***

***Finally, it was observed that surgical treatment using the local bipedicle skin flap technique associated with resection of bony prominences and corrective osteotomies is an effective therapeutic method for the closure of chronic plantar ulcers. It is a reproducible method with excellent results.”***

**Reviewer #3:** This is a well written article that addresses an important issue. However, I do have some comments about the article in its present form.

1. There are a number questions at the start of the article which have not been properly answered. As an example for financial disclosure the response was "NO". The notes clearly state the appropriate words to be used in this section.

***Response: The author(s) received no specific funding for this work.***

2, As above for Data availability. There is no indication of where these data may be accessed.

***Response: These data can be accessed from the author's databases and clinical records at Hospital Geraldo da Rocha, Manaus, Amazonas, Brazil.***

3. Abstract. A total of 234 patients... in contrast 208 patients.....I do not understand what this paragraph means.

***Response: There was a mistake in writing the data. The review was carried out and the appropriate sentence is: “After the surgical treatment, it was verified that the full healing of the wounds occurred in a maximum period of 20 weeks, with an average time of 12 weeks.”***

4. I am confused about the results at the end of page 7 and start of page 8. There are two statistically significant p-values but I am uncertain what was being compared. The statement "The age of the participants were significant variables." is meaningless without a statement of which groups were being compared.

***Response: This confusion has been resolved. Table 3 was renamed to Table 1, which contains the p values of each variable. (quote pages and lines) – Method and Results***

5, Page 8 (last sentence) ... "all 295 feet had complete healing..." .Can the authors confirm that there were no drop outs and all were followed until complete healing. This is an elderly group and I am astounded that all survived and healed with no drop outs.

***Response: Complete healing was assured due to the fact that all patients were hospitalized at Hospital Geraldo da Rocha until the lesions were completely closed, as described in the Methodology. There were no deaths during this period. The maximum period of hospitalization was 20 weeks (5 months).***

6. Table 3. You seem to have 100% recording of all variables used in this study. Is this correct?

***Response: Table 3 has been renamed to Table 1 – and all variables have full data (100%).***

7 Table 3. Please give the statistical method in the table heading. For a correlation it would be helpful to give the correlation coefficients for each p-value.

***Response: Frequency comparison analyzes were performed using Pearson's Chi-square test, with no need for coefficient values.***

8. I presume that Genre means Gender in table 1. Please change

***Response: Gender was modified for Sex. Done.***
