# Supplementary material for: Epidemiological clinical profile and closure of chronic plantar ulcers in patients with leprosy sequelae undergoing orthopedic surgery in a municipality in western Amazon
Source: PLoS One. 2023 Jul 28;18(7):e0284706. doi: 10.1371/journal.pone.0284706 (PMC10381085; doi:10.1371/journal.pone.0284706)
Supplement: Attachment — Submitted filename: Response to Reviewers Plos One.docx [file pone.0284706.s003.docx]

**RESPONSE TO REVIEWERS**

PONE-D-22-04569

EPIDEMIOLOGICAL CLINICAL PROFILE AND CLOSURE OF CHRONIC PLANTAR ULCERS IN PATIENTS WITH LEPROSY SEQUELAE UNDERGOING ORTHOPEDIC SURGERY IN A CITY IN WESTERN AMAZONIA

**Reviewer #1:**

1. Abstract, results, please add more details based on the main findings of this study, for instance, the correlation results.

***Response:*** ***More details were added in the results section based on the main findings of this study and their correlations as described in Page 2, lines 35 to 39***

2. Introduction, this part includes too many paragraphs, please consider merging some and further strengthening the logical structure of this part.

***Response: A few paragraphs were merged on the Introduction and the research’s logical structure was strengthened furthermore as demonstrated in pages 3 and 4; lines 49 to 99.***

3. “Due to the high rate of recurrence of these lesions by conservative methods, several surgeons around the world have reported their techniques with their advances and limitations.”, thus what are the advances and limitations of these techniques? The novelty and significance of this study should further be highlighted.

***Response: The advances and limitations are: Advances in the use of grafts and skin flaps for the success of plantar ulcer closure. Limitations on high relapses rates as a result, as described on page 4, line 79 to 81. The innovation and importance of this study were also highlighted as described on page 4, lines 86 to 99.***

4. Materials and methods, “Patient population and study area”, “Patient recruitment and selection”, could be merged into one part. What were the exclusion criteria?

***Response:*** ***They were gathered in one part: "Patient population and study area", and "Recruitment and selection of patients", as demonstrated on page 5 lines 110 to 117. Exclusion criteria were included according to page 5, lines 118 to 120.***

5. Materials and methods, “Surgical procedures and rehabilitation”, it would be much clearer if the authors explained the surgery process step by step.

***Response:*** ***They were included on the section “Surgical Procedure and Rehabilitation”. The step-by-step section of the surgical procedure as described on page 5, lines 127 to 138.***

6. Materials and methods, please add one more paragraph explaining all measured variables.

***Response: One more paragraph has been added explaining all measured variables as described on page 7, lines 168 to 170.***

7. Results, it is suggested that the authors should include some sub-sessions here for a better description. And please try to condense these contents and only present the main findings here.

***Response: We included sub-sessions for better description of the results and also did some merging highlighting the main findings according to pages 7 and 8, lines 175 to 205.***

8. Discussion, this part also includes too many paragraphs, please consider merging some and further strengthening the logical structure of this part. Moreover, it is suggested that the authors should start by reiterating the main research objectives of this paper, briefly explaining the main findings of this study, and presenting deeper information in this part.

***Response: In this part we merged a few paragraphs as well strengthening furthermore the study’s logical structure. We also reiterate the main objectives of the research, briefly explaining the main findings of the study as found on pages 10 to 12, lines 218 to 283.***

9. In general, please further strengthen the logical structure of this study, and go through the whole manuscript to avoid any grammar mistakes.

***Response: In general, we reinforced the logical structure and highlighted the main objective of the study. We reviewed the manuscript several times correcting grammatical errors as evidenced in the text in pages 2 to 16, lines 22 to 409.***

**Reviewer #3:**

I am concerned by Table 1. There are multiple cells with either no responses or very few responses. It is usual to combine cells when the numbers in each cell are below an acceptable level (say 5 in each cell). Without this, the statistics become meaningless

***Response: Table 1 was adjusted with the p-value and the demonstration of absolute and relative frequency eliminating the cells without answers or with few answers accordingly to page 9, lines 211 to 213.***
