# Supplementary material for: Epidemiological clinical profile and closure of chronic plantar ulcers in patients with leprosy sequelae undergoing orthopedic surgery in a municipality in western Amazon
Source: PLoS One. 2023 Jul 28;18(7):e0284706. doi: 10.1371/journal.pone.0284706 (PMC10381085; doi:10.1371/journal.pone.0284706)
Supplement: Attachment — Submitted filename: Response to Reviewers.docx [file pone.0284706.s004.docx]

**RESPONSE TO REVIEWERS**

PONE-D-22-04569

EPIDEMIOLOGICAL CLINICAL PROFILE AND CLOSURE OF CHRONIC PLANTAR ULCERS IN PATIENTS WITH LEPROSY SEQUELAE UNDERGOING ORTHOPEDIC SURGERY IN A CITY IN WESTERN AMAZONIA

**Journal Requirements**:

**1**- Please review your reference list to ensure that it is complete and correct. If you have cited papers that have been retracted, please include the rationale for doing so in the manuscript text, or remove these references and replace them with relevant current references. Any changes to the reference list should be mentioned in the rebuttal letter that accompanies your revised manuscript. If you need to cite a retracted article, indicate the article’s retracted status in the References list and also include a citation and full reference for the retraction notice.

***Response* Updated and relevant references.**

**Updated and relevant references marked in red are new references and contextualized according to the text data.**

1. WHO. Global update on leprosy (leprosy) [Internet]. 2020 [Accessed November 11, 2021]. Available from: [https://www.who.int/publications-detail-redirect/who-wer9636-421-444. .(Reference Kept](https://www.who.int/publications-detail-redirect/who-wer9636-421-444.%20.(Reference%20Kept%20) )
2. Brazil. Ministry of Health. Health Surveillance Secretariat. Leprosy in Brazil: Characterization of physical disabilities. Brasília - DF, 2020. .( Reference Kept)
3. Souza CDF, Fernandes TRMO, Matos TS, Ribeiro-Filho JM, Almeida GKA, Lima JCB et al. Degree of physical disability in the elderly population affected by leprosy in the state of Bahia, Brazil. Acta Fisiatr . 2017; 24(1): 27-32. .( Reference Kept)
4. Brazil , MS. Manual for the prevention of disabilities. Brasília - DF, 2008.. ( Reference Kept)
5. Singh M, Pawar M. Efficacy of Topical Insulin Therapy for Chronic Trophic Ulcers in Patients with Leprosy: A Randomized Interventional Pilot Study. Adv Skin Wound Care. 2020 Feb;33(2):1-6.. ( Reference updated )
6. Serrano-Coll H & Cardona-Castro N.Neuropathic ulcers in leprosy: clinical characteristics, diagnosis and treatment. J Wound Care . 2022; 31(Sup6): 32-40.( Reference Kept)
7. Riyaz N, Sehgal VN. Leprosy: trophic skin ulcers. Skin Med 2017;15(1):45–51. .( Reference updated )
8. Gahalaut P, Pinto J, Pai GS, Kamath J, Joshua TV. A new treatment for plantar ulcers in leprosy: local superficial flaps. Lepr Rev. 2005;76(3): 220-31. . ( Reference Kept)
9. Price EW. The problem of the plantar ulcer. Lepr Rev. 1964; 35: 267-72. . ( Reference Kept)
10. Srinivasan H, Mukherjee SM: Trophic ulcers in leprosy III. Surgical treatment of chronic foot ulceration. Leprosy India . 1964; 36: 186-193. . ( Reference Kept)
11. Cohen JC, of Miranda ST . Orthopedic Surgical Treatment of Foot in Leprosy. Orthop Clin North Am. 2020; 51(2): 279-91. . ( Reference Kept)
12. Griffts G. Notes on treatment of ulcers in leprosy patients with Polybactrin. Lep Rev. 1966;37(4):227-9. .( Reference updated )
13. Batista KT, Monteiro GB, Y-Schwartzman UP, Aureliano A, Rosa AG, Correia CZ, et al. Treatment of plantar ulcer stemming from leprosy. Rev. Bras. Plastic Cir. 2019; 34: 497-503. . ( Reference Kept)
14. Rao KS, Mahendra Kumar, Oommen PK, Swamy MK, Selvaganapathy S. Collagen leaf and its usefulness in healing ulcers in leprosy patients. Ind J Lepr . 1987; 59: 435-441. . ( Reference Kept)
15. Hu X, Sun H, Han C, Wang X, Yu W. Topically applied rhGM-CSF for the wound healing: a systematic review. Burns. 2011;37(5):729-41. .( Reference updated )
16. Noe JM, Barber J. Chronic leg ulceration in a patient with leprosy. West J Med. 1974;121(5):430-32. .( Reference updated )
17. Cruz H, Kulkarni VN, Dey A, Rendall G. Plantar ulceration in leprosy patients. J Wound Care . 1996; 5(9): 406-11. . ( Reference Kept)
18. Lopes GDF . Efficacy and efficiency of insoles in the prevention and rehabilitation of plantar ulcers in neuropatic feet in leprosy and diabetes [dissertation]. Bauru (SP): Lauro de Souza Lima Institute; 2022. . ( Reference Kept)
19. Noordeen SK, Srinivasan H. Deformity in leprosy: an epidemiological study. Indian J Med Res. 1969; 57(1): 175-81. . ( Reference Kept)
20. GUIMARÃES, Heloísa Cristina Quatrini Carvalho Passos et al. Scientific evidence on leg ulcers as leprosy sequel. Acta Paulista de Enfermagem, v. 32, p. 564-570, 2019. .( Reference updated )
21. Jin Y, Tan Y, Wang J, Zhong H, Yue J, Li H and Yan L. Effect of different surgical methods on leprosy plantar ulcers. Chinese journal of Repairer and Reconstructive Surgery. 2009; 23 (10): 1183-1186. . ( Reference Kept)
22. Reis FJJD, Gomes MK, Cunha AJLAD. Evaluation of the limitation of daily activities and quality of life of leprosy patients undergoing neurolysis surgery for neuritis treatment. Physiotherapy and Research. 2013; 20: 184-190. . ( Reference Kept)
23. Bernardin R, Thomas, B. Surgery for neuritis in leprosy: indications and results of different types of procedures. Lepr Rev. 1997; 68(2): 147-154. . ( Reference Kept)
24. Hidalgo DA, Shaw WW. Anatomical bases of plantar flap design. plastic Reconstr Surg. 1986; 78(5): 627-36. . ( Reference Kept)
25. Yan L, Zhang G, Zheng Z, Li W, Zheng T, Watson JM, Piefer A. Comprehensive treatment of complicated plantar ulcers in leprosy. Chin Med J ( Engl ). 2003; 116(12): 1946-8. ( Reference Kept)
26. Shukla VK, Tiwary SK, Barnwal S, Gulati AK, Pandey SS. Effect of autologous graft cell suspension on chronic wounds that do not heal: a pilot study. Can J Surg. 2010; 53: 6-10. . ( Reference Kept)
27. Nagaraju U, Kashyap P, Raveendra L. Autologous smashed follicular dermal graft with epidermal cell suspension in chronic nonhealing leg ulcers. J Cutan Aesthet Surg. 2020; 13(1): 38-42. . ( Reference Kept)
28. Dheemant M, Yadalla HK, Raju BP. Efficacy of autologous crushed follicular dermal graft and epidermal cell suspension in the treatment of chronic trophic ulcers that do not heal in leprosy patients. Indian Dermatol Online J. 2021; 12(6): 868-872. . ( Reference Kept)
29. Dong L, Li F, Wang Z, Jiang J, Zhang G, Peng J, et al. Techniques for covering soft tissue defects resulting from plantar ulcers in leprosy: Part General considerations and summary of results. Indian J Lepr.1999; 71(3): 285-95. . ( Reference Kept)
30. Santana EMF, Antas EMV, Brito KKG & Silva MA. Profile of leprosy patients in a secondary care center. UFPE Nursing Journal. 2017; 11(11): 4404-9. . ( Reference Kept)
31. Lustosa AA, Nogueira LT, Pedrosa JIS, Teles JBM & Campelo V. The impact of leprosy on health-related quality of life. Rev Soc Bras Med Trop. 2011; 44(5): 621-6. . ( Reference Kept)
32. Upputuri B, Srikantam A, Mamidi RS. Comorbidities associated with non-healing of plantar ulcers in leprosy patients. PLoS Negl Trop Dis. 2020; 14(6): . ( Reference Kept)
33. Pinheiro CIP, Moreira ICCC, Nunez SC, Silva TB, Pereira MS, Campelo DP et al. Clinical-epidemiological profile of people affected by neurotrophic ulcers resulting from leprosy. Res Soc and Develop. 2021; 10(12): . ( Reference Kept)
34. Mustapha G, Obasanya OJ, Adesigbe C, Joseph K, Nkemdilim C, Kabir M, et al. Occurrence of plantar ulcer among leprosy patients in northern Nigeria: a study of contributing factors. Ann Afr Med. Marchar. 2019; 18(1): 7-11. . ( Reference Kept)
35. Ebenso J, Muyiwa LT, Bassey E. Self-care and ulcer prevention groups in Okegbala, Nigeria. Lepr Rev. 2009; 80(2): 187-96. . ( Reference Kept)
36. Cheung JTM, Zhang M. Parametric design of pressure relief foot orthosis using the finite calculation method based on statistics. Physics Eng. Med . 2008; 30(3): 267-77. . ( Reference Kept)
37. Veen NHJ, Mcnamee P. Richards JH, Smith WCS. Cost-effectiveness of interventions to prevent disability in leprosy: a systematic review. plos A . 2009; 4(2): . ( Reference Kept)
38. Tashiro S, Gotou N, Oku Y, Sugano T, Nakamura T, Suzuki H, et al. Relationship between plantar pressure and sensory disorders in leprosy patients - Preliminary research and literature review. Sensors (Basel). 2020; 20(23): 6976. . ( Reference Kept)

References Removed from last version - These references below 09 references have been removed to highlight the above references that are more relevant.

1. Talhari C, Talhari S, Pena GO. Clinical Aspects of Leprosy. Dermatol CLin. 2015; 33: 26-37.
2. Haythornthwaite HW: Closed plaster for trophic ulcers. Leprosy India . 1943; 15: 20-22.
3. Rao KS, Mahendra Kumar, Oommen PK, Swamy MK, Selvaganapathy S. Collagen leaf and its usefulness in healing ulcers in leprosy patients. Ind J Lepr . 1987; 59: 435-441.
4. Pieri FM,Ramos ACV, Crispim JA, Pitiá ACA,Rodrigues LBB, Silveira TRS, et al. Factors associated with disabilities in patients diagnosed with leprosy:a cross-sectional study. hansenology Internacional,2012; 37(12): 22-30.
5. Budel AR, Raymundo AR, Costa CF, Gerhardt C, Pedri LE. Profile of leprosy patients treated at the dermatology outpatient clinic of the Evangelical Hospital of Curitiba. A Bras Dermatol. 2011; 86: 942-6.
6. Batista ES, Campos RX, Queiroz RCG, Siqueira, S. L., Pereira, S. M., Pacheco, T. J., Pessanha, T. O. et al. Socio-demographic and clinicalepidemiological profile of patients diagnosed with leprosy in Campos dos Goytacazes, RJ*. Revista Brasileira de Clinica Médica. 2011; 9(2): 101-6.
7. Miranzi SSC; Pereira LHM & Nunes AA. Epidemiological profile of leprosy in a Brazilian municipality in the period from 2000 to 2006. Rev Soc Bras Med Trop. 2010; 43(1): 62-67.
8. Barreto JG, Bisanzio D, Frade MAC, Moraes TMP, Gobbo AR, Guimarães LS et al. Spatial epidemiology and serological cohorts increase early detection of leprosy. BMC Inf Dis. 2015; 15(1): 1-9.
9. De Oliveira MP, de Sousa JR, de Araujo RS, de Sousa Aarão TL, Quaresma JAS. Protein profile of leprosy patients with plantar ulcer scans from the Eastern Amazon region. Infect dis Poverty . 2017; 6(1): 105.

**2- Additional Editor Comments (if provided):**
Please check the tables as reviewer suggested.

***Response: The table 1 has been revised and it is below***

**3- Reviewer #3**:

Unfortunately, the authors have failed to make any changes to table 1 as I requested, and as such I cannot recommend publication in its present form. To be assured of an appropriate p-value it is recommended that around 5 responses should appear in each cell of the table. I would recommend the following changes.

1. Schooling. Incomplete High school, High School and Higher Education should be combined. While this will not give 5 responses in each cell this will give a more robust p-value

***Response: schooling : incomplete high school, high school and Higher education were joined and added together according to table 1, taking into account the Reviewer's guidelines***

2. Marital status. The Missing option should be excluded from the analysis as this is not a legitimate response.

***Response: Marital status: The option “ Missing was removed from table 1.***

3. Time with ulcer. 31-40, 41-50 and >50 should be combined to give value for >30 and give a reasonable count in all cells.

***Response: Time with ulcer. 32-40 , 41-50 and >50 the data was combined and gave a value of >30***

4. Subscript text should be deleted. There were no confidence intervals generated.

***Response: Subscript text from table 1 was removed.***

5. The main results text may need alteration to take into account the changes to the table.

***Response: Text changed:***

***Summary, Results and Discussions the main results taking into consideration the changes made in table 1 as directed by the Reviewer. Changes observed in the final text.***
